# Boosting the Predictive Power of Virus-Specific T-Cell Clinical Trials via Generative Models

**DOI:** 10.34133/csbj.0208

**Published:** 2026-09-21

**Authors:** Pamela Chansky, Jeremy Rubinstein, Michael Grimley, Stella M. Davies, Emily Blyth, David Gottlieb, Michael A. Pulsipher, Catherine Bollard, Michael D. Keller, Wei Li

**Affiliations:** ^1^ Center for Cancer & Immunology Research, Children’s National Hospital, Washington, DC, USA.; ^2^ Division of Allergy and Immunology, Children’s National Hospital, Washington, DC, USA.; ^3^ The Institute for Biomedical Science, School of Medicine and Health Sciences, George Washington University, Washington, DC, USA.; ^4^ Center for Genetic Medicine Research, Children’s National Hospital, Washington, DC, USA.; ^5^ Department of Pediatrics, University of Cincinnati College of Medicine, Cincinnati, OH, USA.; ^6^ Division of BMT and Immune Deficiency, Cincinnati Children’s Hospital Medical Center, Cincinnati, OH, USA.; ^7^ Blood Transplant and Cell Therapies Program, Westmead Hospital, Sydney, NSW, Australia.; ^8^ Westmead Institute for Medical Research, Sydney, Australia.; ^9^ Sydney Medical School, Faculty of Medicine and Health, University of Sydney, Sydney, NSW, Australia.; ^10^ Division of Pediatric Hematology and Oncology, Intermountain Primary Children’s Hospital, Huntsman Cancer Institute, Spencer Fox Eccles School of Medicine at the University of Utah, Salt Lake City, UT, USA.; ^11^ GW Cancer Center, George Washington University School of Medicine, Washington, DC, USA.; ^12^ Division of Blood and Marrow Transplantation, Children’s National Hospital, Washington, DC, USA.; ^13^ Department of Genomics and Precision Medicine, George Washington University, Washington, DC, USA.; ^14^ University of Maryland Institute for Health Computing, North Bethesda, MD, USA.

## Abstract

The modest number of participants enrolled in phase I/II clinical trials limits the development of accurate models to predict treatment response. This is evidenced by early-phase clinical trials evaluating partially human leukocyte antigen-matched virus-specific T cells (VSTs) to treat virus infections in immunocompromised persons. *In silico* models for VST response are lacking due to the small number of participants enrolled in these clinical trials. Here, we developed a generative artificial intelligence approach to overcoming the small *n* and then trained a binary classifier to predict the likelihood of response to third-party VST therapy. We generated synthetic patient-response training data using a variational autoencoder, whose presence improves the robustness of machine learning models. The binary classification model determines patient response with high accuracy, evidenced by cross-validation using data from 3 third-party VST trials. The combined variational autoencoder and binary classifier provide a potential solution when considering patient suitability for third-party VSTs and also offer a broadly applicable framework to enhance the performance of machine learning models using small-scale clinical datasets.

## Introduction

Early-phase clinical studies, by definition, enroll a relatively limited number of participants, which makes it challenging to build accurate prediction models, and phase I/II trials evaluating the safety and efficacy of virus-specific T-cell (VST) therapy are no exception, often enrolling 100 or fewer patients [1,2]. This “small *n*” problem in the VST field (and other types of novel cellular therapies) has been a major obstacle in building accurate predictive models, which will be critical to stratifying patients and increasing the success rate of clinical trials.

VST therapy is an approach to treating viral infections, which are a common cause of morbidity and mortality in immunocompromised patients [3], especially in patients with inborn errors of immunity (IEIs), such as severe combined immunodeficiency, and in patients receiving T-cell-depleted transplants [4,5]. VST treatment has predominantly been used to treat these patients after allogeneic bone marrow transplant (BMT). In this setting, T cells are isolated from peripheral blood mononuclear cells derived from the patient’s healthy BMT donor or a third-party donor, by either selection or *ex vivo* expansion. The resultant VST product is subsequently infused into the immune-compromised patient either prophylactically or for the treatment of active infections. VST infusion (irrespective of the donor source) after first-line therapy with antivirals is effective in up to 95% of patients, with a 65% to 95% response range reported across multiple studies, with minimal risks of toxicity or graft-versus-host disease (GVHD) [6–8]. Third-party-derived VST products are particularly relevant in the post-solid organ transplant setting [9].

The reasons for the variable patient response rate (RR) are not fully understood. In studying the RR to third-party VST products, multiple patients receiving the same VST formulation achieve different responses, indicating that response is not simply determined by a variation in product quality. Instead, some patient characteristics will likely also impact the response to VST therapy, including their comorbidities, severity of viral infection(s), and human leukocyte antigen (HLA) match with the VST donor, as well as prior and concurrent treatments [7]. To better understand the role of patient characteristics in response, we focus on third-party VSTs. One clinical feature that is likely to affect third-party VST treatment outcome is the concurrent use of immunosuppressive medication at the time of VST therapy. Immunosuppression is known to impair T-cell responses, while reduction of immunosuppression poses a greater risk to the patient including GVHD or graft rejection. Other clinical features that are known to have an association with antiviral outcomes include GVHD, concurrent treatment with corticosteroids, and transplant-associated thrombotic microangiopathy, to name a few [8]. However, even with immunosuppressive medications or any of the previously described considerations, many patients still achieve viral clearance after VST therapy. Therefore, a generalizable primary cause of nonresponse to VST therapy remains unclear [7–10].

A major challenge in treating virally infected patients is deciding whether a patient should receive a VST infusion or another course of antiviral therapy with continued risks of toxicities and antiviral resistance. Because the underlying mechanism behind variable VST RRs is not well understood, the decision to give a VST infusion is not straightforward. A model that can predict patient responses would therefore be highly desirable to identify meaningful clinical features for response and increase the likelihood of success with third-party VST treatment. Such a model has been lacking in part due to the “small *n*” problem in many clinical trial datasets.

Here, we propose a solution to the “small *n*” problem in phase I/II clinical trial datasets using a generative artificial intelligence (genAI) approach. GenAI models have demonstrated great success in natural language processing imaging and video generation [11–13]. Most recently, genAI has been successfully applied to address the issue of limited training data in medical imaging modeling and low-dimensional, tabularized datasets [14,15]. Specific uses in clinical contexts include sepsis prediction and graft failure prediction, among others [16–21]. There are increasing uses of genAI and other modern machine learning approaches to address the “small *n*” problem in other clinical and nonclinical settings [22,23]. Despite these applications, the use of genAI to address the “small *n*” problem in cell therapy clinical trial datasets has been lacking. Our approach uses a variational autoencoder (VAE), an artificial neural network (NN) that takes input data and produces new data that share the same distributions as the original data. VAE consists of both an encoder and a decoder, where the encoder scales down the original data, encoding these into a lower-dimensional representation (latent space), and the decoder rescales the data, reconstructing the dimensionality of the original dataset [24,25]. We demonstrated that our VAE approach generates synthetic data whose structure is highly similar to the original data and boosts the performance of a machine learning binary classifier that predicts patient responses to VST therapy. Importantly, our binary classifier was validated using data from 2 other independent third-party VST cohorts, demonstrating its applicability across different cohorts.

**Fig. 1.**
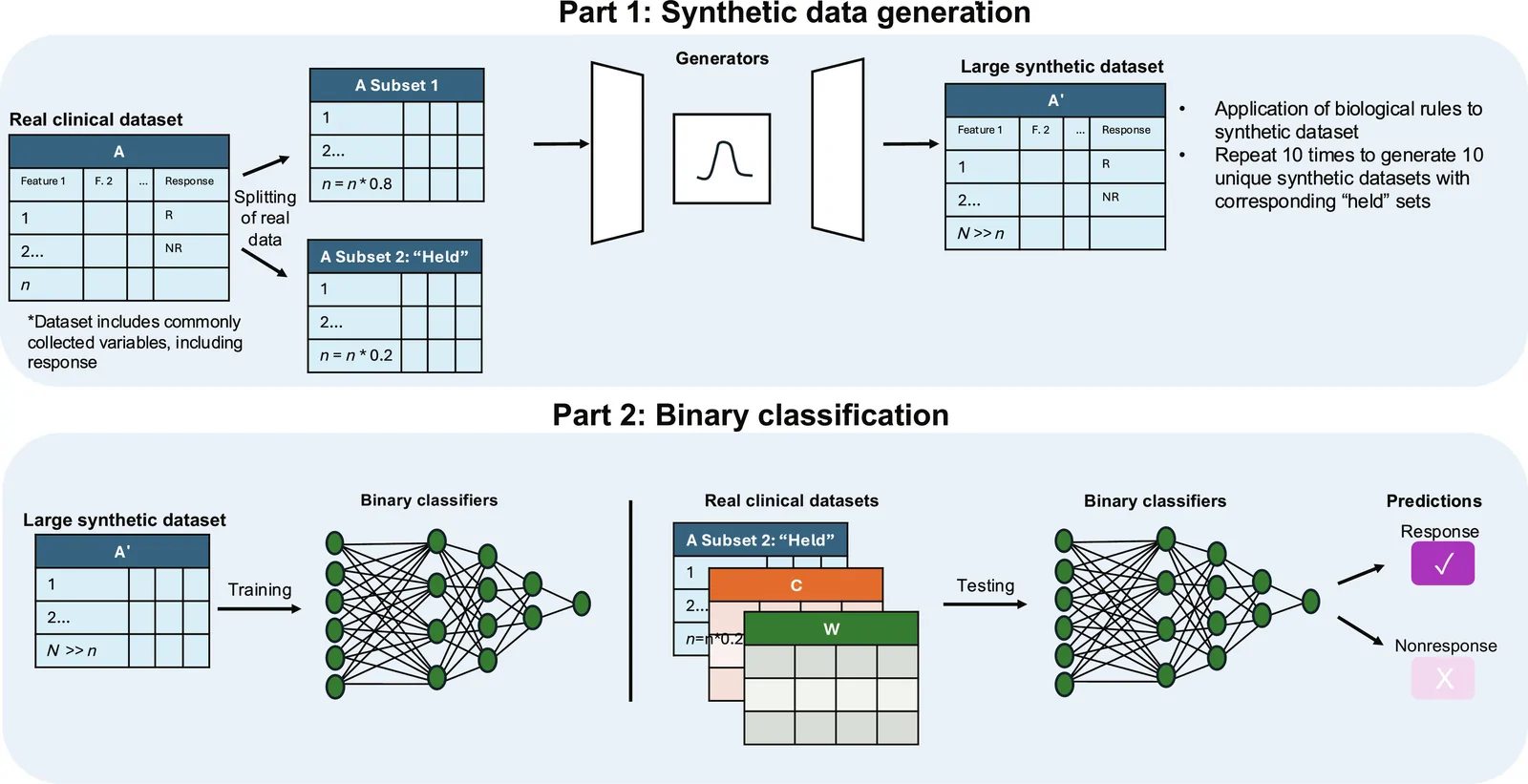
Overview of the deep learning model structure. Visualization of the use of clinical data as input to a simplified variational autoencoder, where the data are encoded, sampled from the latent representation, and then new data are decoded into a synthetic dataset matching the structure of the original data. Synthetic data are then used as in the input to binary classifiers, which are trained on a subset of the data and tested on the withheld clinical data, predicting response (R) or nonresponse (NR).

There is increasing interest in applying computational techniques, originally designed for nonclinical problems, to clinical studies. One study suggested the hypothetical use of a VAE for increasing sample sizes without having to increase patient recruitment to clinical studies [24]. Deep learning models have been used to solve classification problems in the clinical space for decades, with specific applications in sepsis prediction, graft rejection prediction, GVHD risk prediction, chimeric antigen receptor T-cell (CAR-T) toxicity prediction, and MRI interpretations [14,16–21]. Specifically, multilayer sequential models, consisting of dense layers with varying activation functions can be used to model the relationships between multivariable inputs to make predictions [26]. While these approaches are often used for highly dimensional data (and here we have approximately 40 variables), they can also be used when the underlying relationships between the input variables are unknown or complex [25–28]. Given the lack of definitive data for specific variables and VST response, it is appropriate to utilize a deep learning approach for the classification of response and nonresponse. In turn, we directly apply a VAE approach to increase the sample size from a third-party VST therapy clinical study to enable stronger statistical analyses and computational modeling results (Fig. 1).

## Materials and Methods

### Data

Metadata from the Antiviral Cellular Therapy for Enhancing T-Cell Reconstitution before or after Hematopoietic Stem Cell Transplantation (ACES) clinical trial (NCT03475212) as well as the Allogeneic Virus-Specific T Cell Lines (CHAPS) trial (NCT02510417), which included 74 pediatric third-party VST infusions during the years 2017 to 2020, are used for the primary modeling experiments (“ACES dataset”) [8]. Patient age ranges from 5 months to 45 years, with a median age of 11.5 years. Two additional third-party VST infusion datasets are used for testing, the Cincinnati Children’s Hospital cohort (i.e., “Cincinnati dataset”) and the Westmead Hospital cohort (“Westmead dataset”) [29,30]. The Cincinnati dataset includes 118 VST infusion records, with patients ranging in age from 1 month to 73.6 years, with a median age of 15.2 years. The Westmead dataset contains 49 VST infusion records, with patients ranging in age from 1 to 71 years, with a median age of 60 years.

The variables included in the dataset are continuous, categorical, or Boolean valued. Categorical data are one-hot encoded prior to modeling. Continuous variables are log normalized. Variables include type of viral infection (adenovirus, Epstein–Barr virus [EBV], and cytomegalovirus [CMV]), log-normalized viral load at time of infusion, original diagnosis (IEI, malignant, and nonmalignant hematology condition), preparatory category (myeloablative, reduced conditioning, and unknown), transplant donor (mismatched related donor, mismatched unrelated donor, matched unrelated donor, matched related donor, umbilical cord transplant, and other—predominantly autologous), cellular depletion (alpha-beta T-cell receptor CD19 depletion [abTCR CD19]), and receipt of immunosuppressive medications at the time of infusion, including treatment with systemic corticosteroids, budesonide, tacrolimus (FK), cyclosporine (CsA), mycophenolic acid (MMF), sirolimus, anti-thymocyte globulin (ATG), and alemtuzumab (Campath); use of antivirals prior to infusion, including ganciclovir, valganciclovir, foscarnet, cidofovir, brincidofovir, and rituximab; and degree of HLA match between the patient and the VST product. The degree of HLA match ranges from 1 to 6, based on the number of major alleles shared. The viral load is measured from blood samples by polymerase chain reaction in international units per milliliter with relevant conversions for each virus type, followed by a log normalization. An unknown prep category indicates that the patient’s prep category for their BMT infusion was missing in the dataset. Several of these variables and their relationships to each other, i.e., treatment with valganciclovir for CMV infections in the posttransplant setting, have been well studied [31–33]. Missing data were handled as follows: variables that were not collected across all 3 cohorts were excluded rather than imputed, and where a categorical variable was missing for an individual record, the missing value was retained as an explicit category, for example, the “unknown” preparative regimen category, rather than imputed. Reporting of the classification models follows the TRIPOD+AI recommendations where they apply to this study design.

### Generative modeling for data synthesis (VAE, GAN, and GC)

All models were built in Python 3.10.4; version information for relevant packages is included in the Supplementary Materials.

To begin training the generative models, we utilized the ACES records, of which 80% were converted to tensors and were further split into training and testing cohorts for the generative models, such that 64% of the original records were used for training the generative models and 16% were used for testing the generative models. For example, the ACES cohort contains 74 records, so 48 records were used for training the generative models, 12 were used for testing the generative models, and an additional 14 were not seen by any portion of the generative models and comprised the “withheld records” portion. The withheld records were used later in testing the binary classifiers.

We tested VAE, generative adversarial network (GAN), and Gaussian copula (GC) structures for data generation [34–39]. The primary VAE, GAN, and GC models each generated 5,000 synthetic records over 1,000 training epochs. In addition, we ran the VAE to generate 500 records and 50,000 records and tested the data fidelity and utility in each case. After generation, data were subjected to removal if they did not meet biological requirements. For example, if a synthesized record returned 1 (true) for ATG and 1 (true) for alemtuzumab, it was removed from the synthetic dataset. This is because a real patient is highly unlikely to be administered both drugs concurrently. Records were also removed if they returned 1 (true) for multiple diagnostic categories or if they returned 1 (true) for multiple BMT donor types. The generative models were cross-validated; the training and testing process was repeated 10 times, with a random selection of the ACES cohort being utilized as input to the generative models each time (and a corresponding selection of the ACES cohort being withheld for later testing). Thus, 10 synthetic datasets were generated from each model, with 10 corresponding sets of withheld ACES records.

We employed the same method of training the VAE model when using the Cincinnati and Westmead datasets, where again 80% of the records were used as input for the VAE model and 20% were withheld for downstream testing of the classifier model(s). We additionally created a dataset consisting of all 3 datasets combined and used this as the “Combination” dataset for VAE training, where, again, 64% of the records were using for training the VAE, 16% were used for testing the VAE, and the remaining 20% were withheld for testing the binary classifier.

The GAN and GC were optimized by hyperparameter tuning, with the generator and discriminator learning rates tuned in the GAN and the default distribution for the categorical variables tuned in the GC. The VAE was further optimized by altering the number of layers and nodes in the structure.

To assess synthetic data fidelity, we calculated similarity scores, comparing the distribution of a single variable in the ACES cohort to the distribution of that same variable in the synthetic cohort. For continuous variables, the Kolmogorov–Smirnov complement was used as the similarity score, and for binary variables, the total variation distance complement was used as the similarity score [40,41]. In both cases, higher similarity scores indicate more similar distributions, i.e., synthetic variables with distributions that are more similar to their real counterparts. Similarity scores are displayed as box-and-whisker plots for each of the 10 datasets. To assess intervariable relationships, the Spearman correlation between each variable was calculated in each dataset, and the average results are displayed in a heatmap [42]. The Hellinger distances were calculated as another metric of individual variable distribution similarities, and smaller values indicate more similar distributions [43].

### Classification

For the purposes of this model, response is encoded as 0 for nonresponse or 1 for response. Responses include both partial and complete responses, where a complete response is typically indicated by viral load dropping to 0 IU/ml or an undetectable level and a partial response is indicated by a decrease in the measurable viral load compared to the pre-infusion viral load. The custom binary classifier, NN, consists of a convolutional layer with a kernel size of 4, a dense layer with a rectified linear unit activation, a dropout rate of 0.2, and a final dense layer with sigmoid activation. The classification model is trained on the synthetic data, for 30 epochs, and the model is tested on the corresponding withheld real records, as described in the “Generative modeling for data synthesis (VAE, GAN, and GC)” section, following a train on synthetic test on real method. The process is repeated for all 10 synthetic datasets, generating average results across each dataset.

To assess performance of all binary classifiers, the area under the receiver operating characteristic curve (ROC-AUC), accuracy, recall, and precision are calculated. Results are compared across the 9 classifiers, with tuning of relevant hyperparameters by randomized search using 2 to 4 values for each hyperparameter search space. The tuned hyperparameters for each model were as follows: logistic regression classifier: penalty type, tolerance, C, and solver type; random forest classifier: loss criterion and max features; naïve Bayes classifier: variance smoothing; *k*-nearest neighbors classifier: number of neighbors and leaf size; support vector machine: C, kernel, and tolerance; gradient boosting classifier: loss type, learning rate, and number of estimators; light gradient boosting machine (LGBM): minimum number of child samples and minimum child weight; and XGBoost classifier: max depth and number of estimators. Because the Cincinnati and Westmead cohorts are class imbalanced, precision–recall curves are also reported alongside ROC-AUC.

The same methods of testing are used for assessment of the best model, the NN, on the Cincinnati and Westmead datasets. After testing the utility of the ACES-trained-VAE-synthesized data in the classification task, we also use the same method of testing the utility of Cincinnati-trained-VAE-synthesized data, Westmead-trained-VAE-synthesized data, and the combination-trained-VAE-synthesized data.

We additionally assess the utility of using synthetic data by training and testing the model on nonsynthetic data only, where the same best model structure is used and is trained over 30 epochs.

We test the use of a foundation model optimized for small data, TabPFN [15]. We train the model on a portion of one clinical dataset, e.g., 80% of the ACES records, and then test the model on the remaining data, e.g., the other 20% of the ACES records and the Cincinnati and Westmead datasets. This is repeated such that each clinical dataset is used for training. To enable usage of this model, we engage Python 3.11.6. The model is assessed using accuracy, recall, and precision.

### Feature importance

In addition to the model’s performance, we also assess the feature importance. We use the Shapley factors for feature contributions, where a larger Shapley value indicates greater predictive quality [44,45]. Shapley values are calculated for the NN trained on ACES-trained-VAE-synthesized records and for the nonsynthetic ACES records, where corresponding withheld ACES records were utilized for model testing. We utilize the Shapley method due to its relevance in the machine learning research community.

## Results

### Response trends across datasets

**Fig. 2.**
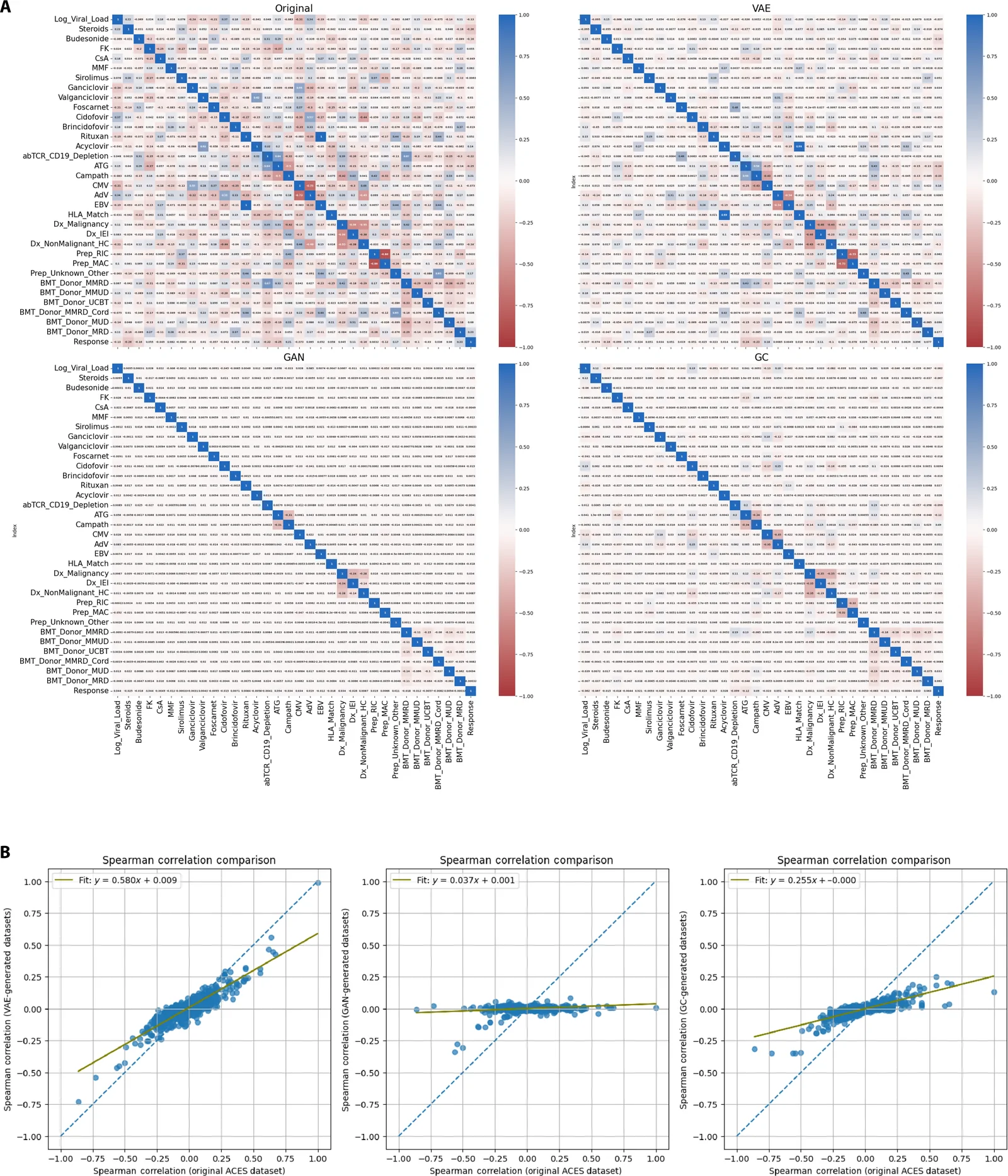
Correlations between variables in the variational autoencoder (VAE)-synthesized dataset are similar to the correlations between variables in the original dataset. Correlation is measured using the Spearman method. (A) More positive values are blue, and more negative values are red; low absolute values (i.e., near 0) are whiter. From top left to bottom right: “Original” refers to the ACES dataset; “VAE”, to the variational autoencoder (VAE)-generated dataset; “GAN”, to the generative adversarial network (GAN)-generated dataset; and “GC”, to the Gaussian copula (GC)-generated dataset. (B) Comparing the Spearman correlation values calculated between each pair of variables in the ACES dataset versus the VAE-generated dataset, GAN-generated dataset, and GC-generated dataset. ACES, Antiviral Cellular Therapy for Enhancing T-Cell Reconstitution before or after Hematopoietic Stem Cell Transplantation trial.

We evaluated 3 VST clinical trial datasets, referred to as “ACES”, “Cincinnati”, and “Westmead”, from 3 distinct clinical trials using third-party VSTs that are either completed or ongoing [8,29,30]. Clinical RRs to VST infusions varied across the 3 datasets as follows: ACES RR was 66% (56 patients), Cincinnati RR was 88% (76 patients), and Westmead RR was 97% (30 patients). Response to a VST infusion was based on specific protocol definitions, including a minimum 1-log fold decrease in viral load at 28 d post-infusion, as measured via polymerase chain reaction (Table 1). Details of viral load measurements and normalization are provided in Materials and Methods. The maximum mean discrepancies between the 3 datasets are 0.0346 (ACES and Cincinnati), 0.0465 (ACES and Westmead), and 0.0508 (Westmead and Cincinnati). We assessed the correlations between individual variables and response in each of the 3 datasets. The variables with the strongest positive correlations with response in the ACES dataset were BMT donor type matched related (0.23), treatment with alemtuzumab (Campath) (0.21), unknown prep category for transplant (0.17), treatment with acyclovir (0.14), and an original diagnosis of IEI (0.11) (Fig. 2). In the Cincinnati dataset, the top positive correlators with response were CsA (0.22), treatment with sirolimus (0.1), treatment with MMF (0.11), treatment with budesonide (0.10), and having an EBV infection (0.10). In the Westmead dataset, the top positive correlators with response were treatment with foscarnet (0.20), treatment with MMF (0.20), treatment with tacrolimus (FK, 0.19), HLA match (0.17), and having a CMV infection (0.17) (Fig. S1).

**Table 1.** Patient demographics

Criteria	Value		
	ACES	Cincinnati	Westmead
Infusion count	74	118	49
Patient count (below values are per patient)	56	76	30
Median age (range)	12.1 years (5 months–45 years)	15.2 years (1 month–73.6 years)	60 years (1–71 years)
Underlying diagnosis			
Malignancy	25 (44.6%)	38 (50.0%)	25 (83.3%)
Inborn error of immunity	18 (32.1%)	22 (28.9%)	5 (16.7%)
Other nonmalignant hematologic condition	13 (23.2%)	3 (3.9%)	0 (0%)
HSCT donor types			
MMRD	19 (34.0%)	20 (26.3%)	5 (16.7%)
MMUD	7 (12.5%)	19 (25.0%)	2 (6.7%)
None	0 (0.0%)	0 (0%)	0 (0%)
UCBT	8 (14.3%)	0 (0%)	1 (3.3%)
MMRD + Cord	2 (3.6%)	0 (0%)	0 (0%)
MUD	12 (21.4%)	31 (40.8%)	16 (53.3%)
MRD	8 (14.3%)	5 (6.6%)	6 (20%)
abTCR depletion	10 (17.9%)	4 (5.3%)	0 (0%)
Targeted virus			
CMV	25 (44.6%)	45 (59.2%)	28 (93.3%)
AdV	32 (57.1%)	30 (39.5%)	1 (3.3%)
EBV	5 (8.9%)	15 (19.7%)	1 (3.3%)
Prep category			
RIC	20 (35.7%)	21 (27.6%)	19 (63.3%)
MAC	33 (58.9%)	20 (26.3%)	11 (36.7%)
Other (includes BEAM)	2 (3.6%)	29 (38.2%)	0 (0%)
Response	37 (66.1%)	67 (88.2%)	29 (96.7%)
Medications (immediately prior to or concurrent with an infusion)			
Steroids	19 (33.9%)	38 (50.0%)	7 (23.3%)
Budesonide	3 (5.4%)	5 (6.6%)	0 (0%)
FK	26 (46.4%)	31 (40.8%)	5 (16.7%)
CsA	6 (10.7%)	16 (21.1%)	17 (56.7%)
MMF	4 (7.1%)	5 (6.6%)	6 (20%)
Sirolimus	5 (8.9%)	6 (7.9%)	0 (0%)
Ganciclovir	12 (21.4%)	19 (25.0%)	14 (46.7%)
Valganciclovir	4 (7.1%)	7 (9.2%)	2 (6.7%)
Foscarnet	7 (12.5%)	19 (25.0%)	6 (20%)
Cidofovir	28 (50.0%)	11 (14.5%)	2 (6.7%)
Brincidofovir	8 (14.3%)	4 (5.3%)	0 (0%)
Rituxan	5 (8.9%)	4 (5.3%)	0 (0%)
Acyclovir	1 (1.8%)	0 (0%)	0 (0%)
ATG	23 (41.2%)	20 (26.3%)	0 (0%)
Campath	17 (30.4%)	24 (31.6%)	0 (0%)

HSCT, hematopoietic stem cell transplantation; MMRD, mismatched related donor; MMUD, mismatched unrelated donor; UCBT, umbilical cord transplant; MUD, matched unrelated donor; MRD, matched related donor; abTCR, alpha-beta T-cell receptor; CMV, cytomegalovirus; AdV, adenovirus; EBV, Epstein–Barr virus; RIC, reduced-intensity conditioning; MAC, myeloablative conditioning; FK, tacrolimus; CsA, cyclosporine; MMF, mycophenolic acid; BEAM, carmustine (BCNU), etoposide, ara-C, melphalan; ATG, anti-thymocyte globulin

The strongest negative correlators with response in the ACES dataset were treatment with steroids (−0.26), treatment with ATG (−0.25), abTCR CD19 depletion (−0.22), an original diagnosis of nonmalignant hematologic condition (−0.16), and treatment with budesonide (−0.16). The top negative correlators with response in the Cincinnati dataset were treatment with brincidofovir (−0.22), treatment with ATG (−0.19), log viral load at the time of infusion (−0.18), an original diagnosis of nonmalignant hematologic condition (−0.14), and treatment with Campath (−0.10). The top negative correlators with response in the Westmead dataset were having an EBV infection (−0.30), log viral load at infusion (−0.20), BMT donor type matched unrelated donor (−0.16), treatment with CsA (−0.06), and an original diagnosis of malignancy (−0.05). The negative correlations with response drop off in strength much more quickly in the Westmead (adult) dataset than in the 2 pediatric datasets. Of note, the log-normalized viral load has a negative correlation with response in all 3 datasets, indicating that as the viral load at the time of infusion increases, the likelihood of response to VST therapy decreases (Fig. S1).

### Generation of synthetic VST infusion records: VAE, GAN, and GC models

To overcome limitations in predictive power due to a small input sample size, we first employed generative methods to increase the sample size for the downstream classifier. We tested several methods for data synthesis, most notably a VAE, a GAN, and a GC synthesizer [34–39]. To train and evaluate the 3 methods, a 10-fold cross-validation was performed as described in Materials and Methods. After data splitting and preparation, 80% of the remaining VST infusion records (64% of the initial records) were used as the input to each of the generative models and 5,000 synthetic VST infusion records were generated. Of note, the response variable is included in the datasets and is also generated. After data generation, we assessed the synthetic datasets’ similarities to the corresponding real VST infusion dataset (ACES), using the Spearman correlations, total variation distance complement, Kolmogorov–Smirnov complement, and multivariate Hellinger distance.

In comparing the intervariable relationships, the VAE is best able to reproduce correlations seen in the ACES dataset. The GAN and GC models fail to reproduce some of the strong correlations identified in the original ACES dataset that are upheld in the VAE-generated dataset, e.g., the correlation between the prep categories of reduced-intensity conditioning and myeloablative conditioning. In the ACES dataset, the Spearman value is −0.86; in the VAE dataset, it is −0.73; in the GAN dataset, it is −0.0065; and in the GC dataset, it is −0.32 (Fig. 2). Thus, the VAE-generated data have intervariable correlations most similar to those in the real data when compared to the GAN- and GC-generated data. VAEs and GANs are both based on an NN structure, which may be a better fit for our data than the GC structure. However, GANs can be more complicated to train, which may be why the VAE appears to more successfully generate clinical records when compared to the GAN [46].

The majority of VAE-synthesized data are usable and represent possible patients, with on average 75.7% of the records representing possible patients. A possible patient is any record that contains data that do not break any biological or medical rules and therefore represents a patient that could in theory exist. If a record breaks a known biological rule, i.e., if a single record contains true values for both treatment with alemtuzumab and treatment with ATG or if a single record contains true values for multiple BMT donor types, these records are considered impossible patients and are excluded from the training and testing of the subsequent classification model.

**Fig. 3.**
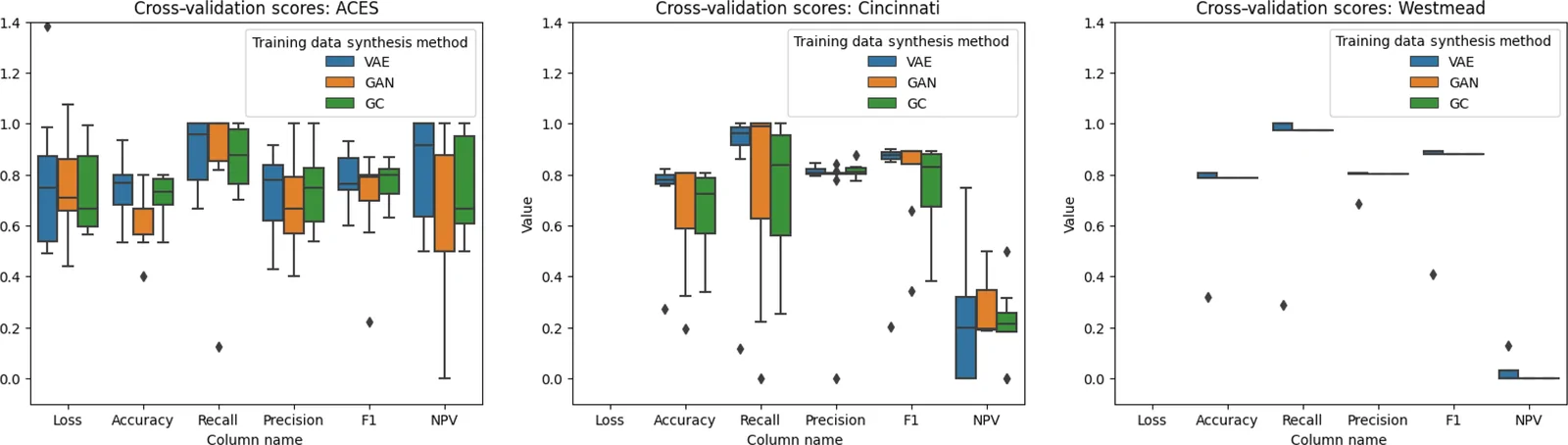
Training a predictive classifier on variational autoencoder (VAE)-generated data results in improved performance compared to training on generative adversarial network (GAN)-generated data. Results of training the custom neural network (NN) classifier on ACES-trained-VAE-synthesized data versus ACES-trained-GAN-synthesized data and tested on corresponding withheld ACES, Cincinnati, and Westmead records. Box-and-whisker plots displaying the results from 10-fold cross-validation for loss (binary cross-entropy), accuracy, recall, precision, F1, and negative predictive value. The legend indicates which synthesis method was used to generate the relevant training data for the NN, and each method is labeled with a different color. ACES, Antiviral Cellular Therapy for Enhancing T-Cell Reconstitution before or after Hematopoietic Stem Cell Transplantation trial.

We later assessed the utility of VAE-, GAN-, and GC-generated synthetic data by training a classifier on each of the synthetic datasets and comparing the accuracy metrics (Fig. 3). In addition to improved data fidelity, the VAE also had improved data utility, wherein the downstream NN classifier had higher median test performance metrics when trained on VAE-generated data than when trained on GAN- and GC-generated data.

Additionally, the quantity of data generated was validated by generating varying quantities of records and using the synthetic data to train the same structure of classifier; 500, 5,000, and 50,000 records were generated and used for downstream modeling. Similar accuracy performance scores were achieved for all 3 quantities of data used (Fig. S2).

### Further optimization of the VAE structure

The VAE was selected as the most suitable generative method for our data; we next determined the optimal VAE structure for modeling a small dataset by changing the number of layers and number of nodes. To do so, we tested 9 combinations of varying number of layers and nodes. To assess the performance of each model, the similarity scores were averaged across all variables to get a single value. All 9 models had median similarity scores over 0.7, likely due to the nature of generating binary variables. However, the distribution of the model scores vary, where 2 models using the smallest number of nodes—2 models with the fewest number of layers and 2 models with the largest number of layers—both have large ranges in similarity scores (nearly 0.5). The model with the most layers and most nodes had less variation than most small models; however, this model also had several outliers with similarity scores below 0.6. The best-performing models were those with a medium number of layers and large or medium number of nodes. Both had median similarity scores over 0.9 and lower quartiles over 0.8, with only 2 outliers below 0.8 (Fig. S3). We selected the model with a medium number of layers and nodes, because the increase in performance between the medium and large models was minimal and larger models are generally more computationally expensive to train.

### Prediction of third-party VST therapy clinical response: Binary classifier models

After generating sufficient high-fidelity synthetic pre-infusion data, we proceeded with the binary classification problem. With an input of 33 pre-VST infusion variables, i.e., features that are collected about a patient prior to their VST infusion, we trained each binary classifier to predict the likelihood of response or nonresponse to VST therapy. After training on ~3,700 synthetic records over 30 epochs, we tested the models on the corresponding sets of withheld records. We trained exclusively on synthetic data and tested on real data to ensure that there was no data leakage between the training and testing sets. The subset of the real datasets used for testing the binary classifiers was not seen by the generative models or by the binary classifiers prior to testing. Our custom NN achieved over 70% F1-scores when tested on the withheld ACES, Cincinnati, Westmead, and combination datasets.

**Fig. 4.**
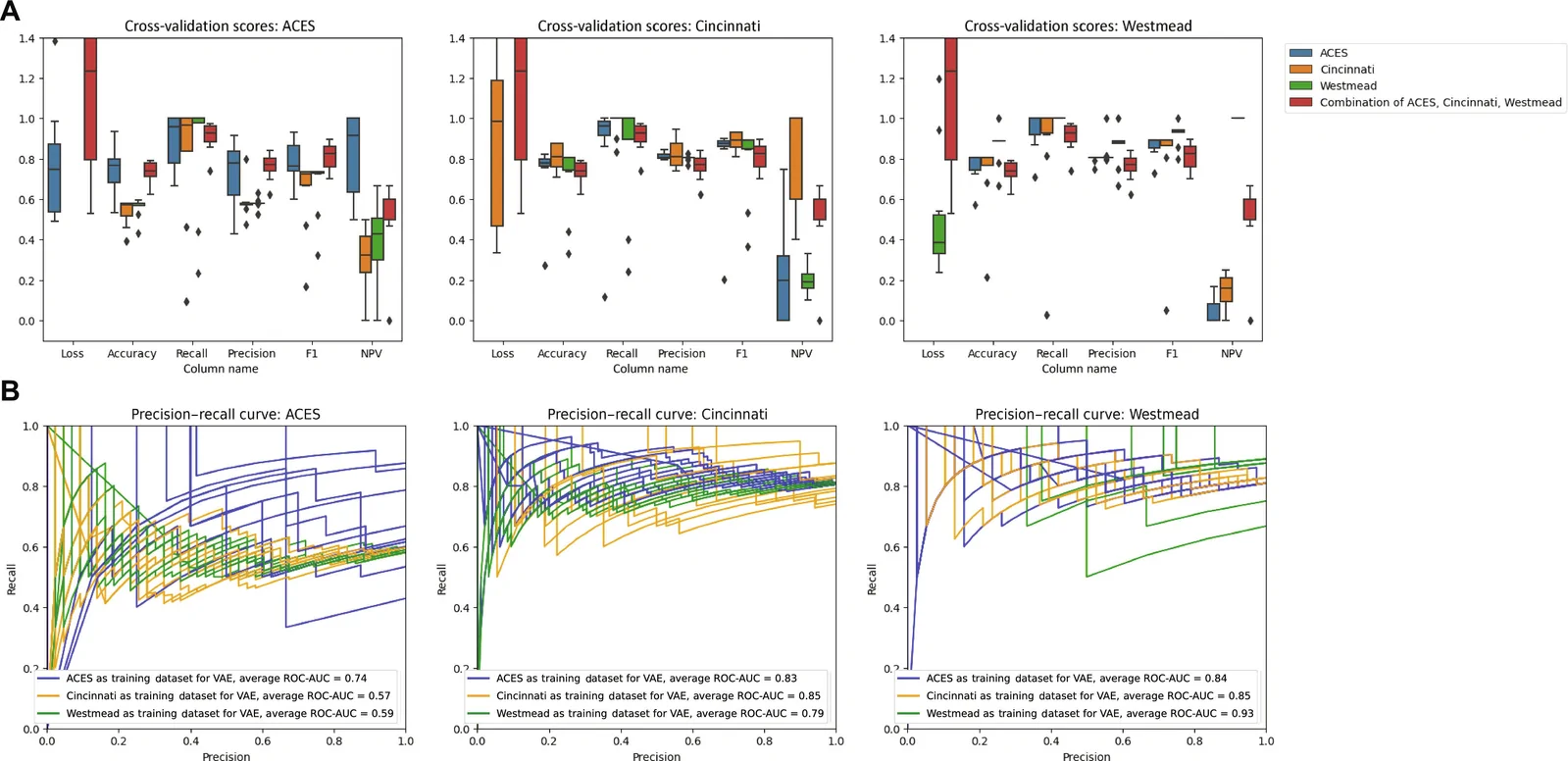
The custom classifier predicts virus-specific T-cell (VST) response with high accuracy, recall, precision, and F1-scores. Results of training the custom neural network classifier on variational autoencoder (VAE)-synthesized data and tested on corresponding withheld ACES, Cincinnati, and Westmead records. (A) Box-and-whisker plots displaying the results from 10-fold cross-validation for loss (binary cross-entropy), accuracy, recall, precision, F1, and negative predictive value. The legend indicates which dataset was used to train the VAE, and each dataset is labeled with a different color. (B) Precision–recall curves of testing the custom neural network classifier on the corresponding ACES, Cincinnati, and Westmead datasets. The legend indicates which dataset was used to train the VAE, and each dataset is labeled with a different color. Values reported in the legend are the mean area under the receiver operating characteristic curve (ROC-AUC) across the 10 cross-validation folds. ACES, Antiviral Cellular Therapy for Enhancing T-Cell Reconstitution before or after Hematopoietic Stem Cell Transplantation trial.

To assess the binary classifier’s applicability outside of a single dataset, we tested the NN classifier’s performance on independent datasets, meaning that when the generative model was trained on a portion of the ACES data, we performed testing of the binary classifier on the withheld ACES records as well as on the other 2 datasets, Cincinnati and Westmead. Of note, most records in the Cincinnati and Westmead cohorts are responders. When class imbalance occurs, a more popular metric is the precision–recall curve, which is included and shows promising results for these datasets. Within each dataset, the classifier achieves over 60% median F1-scores (Fig. 4). The classifier’s ability to accurately predict the likelihood of response in multiple unseen datasets indicates its viability.

We additionally tested several standard classifier methods, a logistic regression model, a random forest model, a naïve Bayes model, a *k*-nearest neighbors model, a support vector machine, a gradient boosting classifier model, an LGBM model, an XGBoost model, and a custom NN. The NN scores the highest median accuracy, precision, and recall on the ACES withheld data and also scores over 0.7 in all 3 metrics in the Cincinnati and Westmead datasets. The NN also has the lowest loss values compared to the other models tested (Fig. S4). Of note, the LGBM model did not result in measurable performance when tested on the external datasets.

To assess the utility of the synthetic data, we also trained the NN on nonsynthetic data. We split the original ACES dataset into a training and a testing cohort and used the same NN structure. The median values for the accuracy, precision, recall, and F1-score metrics were the same or better in all tested datasets when the model was trained on VAE-synthesized data compared to nonsynthetic data. The only metric that underperformed when using the synthetic data compared to nonsynthetic training data was the AUC when tested on the Westmead dataset; however, both models had acceptable AUCs of 0.84 and 0.87 (Fig. S5A and B).

### Feature importance

**Fig. 5.**
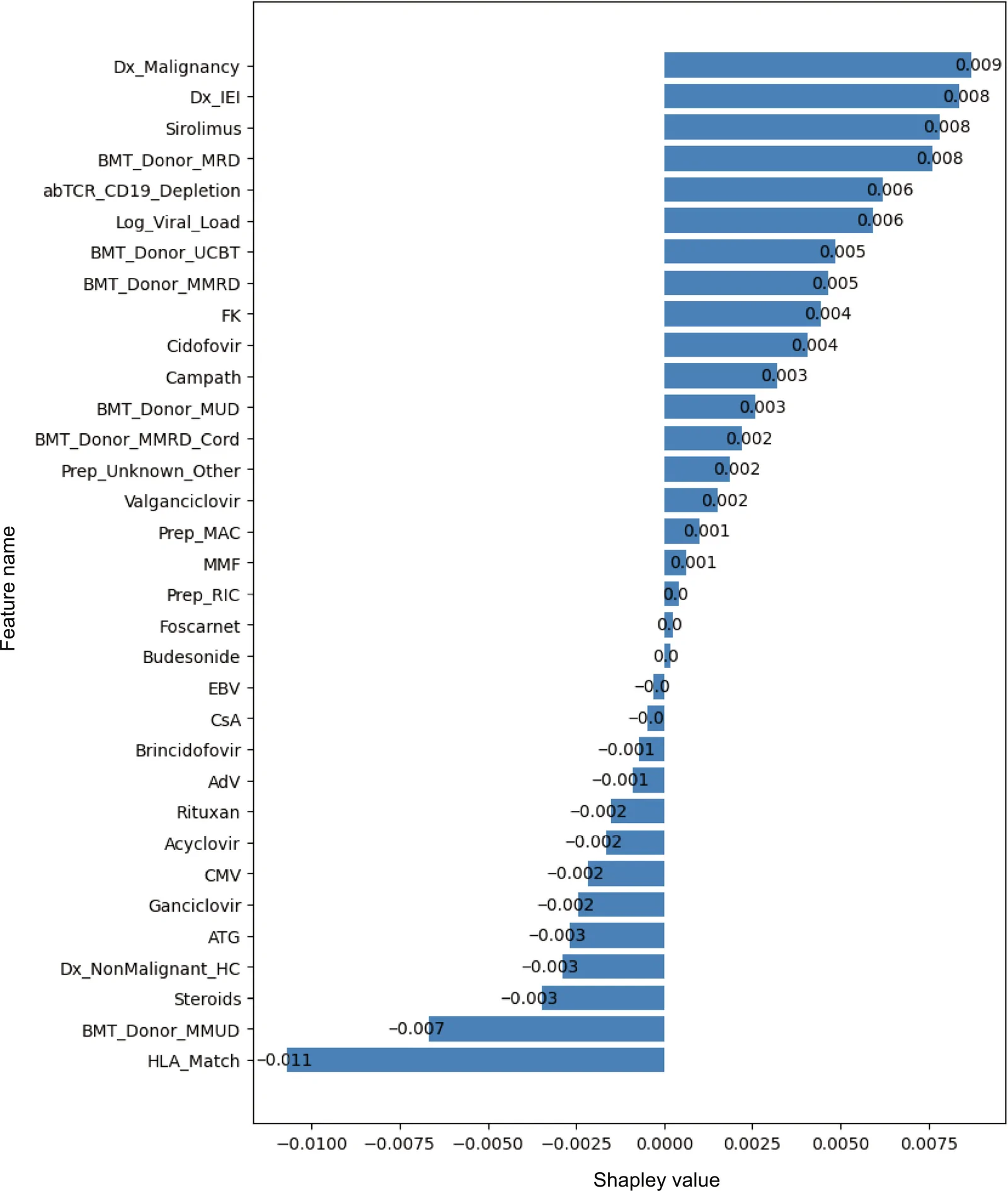
All features have relatively insignificant Shapley values. The Shapley value is displayed in a bar plot for each feature. Values range from 0.009 to −0.011, where larger absolute values indicate a greater contribution to the model’s predictive ability.

In our evaluation of the best-performing model, the NN, we also considered feature importance, with the goal of identifying if any individual variables were contributing most to the model’s predictive ability. No individual features had large Shapley values, indicating the model’s use of all features to generate predictions. We identified the top positive contributors as an original diagnosis of malignancy, an original diagnosis of IEIs, treatment with sirolimus, a matched related donor for BMT, and undergoing abTCR CD19 depletion. The top negative contributors were the number of matching HLA alleles, a mismatched unrelated BMT donor, treatment with steroids, an original diagnosis of hematologic condition, and treatment with ATG. While none of the variables had a strong correlation with response in the clinical datasets using Spearman values, the Shapley values indicate their value in patient stratification, where a matched related donor for BMT, for example, has a positive predictive value and a small positive correlation with response in the ACES dataset (Fig. 5).

Interestingly, when training the NN on nonsynthetic data only, we found that several features had opposite predictive values when compared to the NN trained on synthetic data. For example, undergoing abTCR CD19 depletion has a positive predictive value in the NN trained on synthetic data but has a negative predictive value in the NN trained on nonsynthetic data (Fig. 5 and Fig. S5C). At the same time, other features share their Shapley trends when comparing the NN trained on synthetic *vs* nonsynthetic data; e.g., an original diagnosis of malignancy remains the top positive Shapley value in both models. Of note, there are substantially more records used for training and testing when utilizing the synthetic data compared to nonsynthetic data, and all Shapley values are very small regardless of training data type. This variability further indicates that no one variable has an obvious relationship with the ability to predict response, and the quantity of data may play a role in the model’s ability to learn this relationship.

## Discussion

Choosing the right treatments for individual patients is critical to enhancing clinical outcomes. For VST therapy, the decision of patient selection for third-party VST treatment versus continuing with additional course(s) of antiviral therapy can be challenging. The lack of large datasets, complex underlying relationships between clinical variables, and variations in how response may be determined all contribute to the difficult nature of this problem. In this study, we presented a novel dual-modeling framework, where genAI was first used to generate accurate synthetic clinical data, followed by training a binary classification model to predict patient response using these synthetic records. We have shown that a VAE approach can generate data that are highly similar to real data in terms of individual variable distributions and intervariable relationships (e.g., mutual exclusiveness). A machine learning model can then be trained on the synthetic data to accurately predict the likelihood of therapeutic response.

In selecting the VAE as the best generative method for this problem, we acknowledge that there are still potential opportunities to further improve the generative approach. The rationale for this stems from the fact that when applying the biological rules as a filter, we remove approximately 24.3% of generated records. This implies that the VAE itself has not perfectly learned all relevant relationships between variables and could therefore benefit from further optimization. However, the benefit of using the synthetic data to train the binary classifier is evidenced by the improved performance when compared to training on nonsynthetic data. This is likely due to the increase in training size as well as the “loosened” distribution metrics, which increase the diversity of the training data, enabling better performance on the other datasets that have slightly different feature distributions. We also acknowledge that the records that break biological rules, which represent a form of hallucination, may not necessarily cause large changes in the binary classifier performance if they were to be included, given that these are not the majority of the records. However, we remove them to avoid introducing unnecessary false information to the model and improve the interpretability and trustworthiness of the binary classifier. We compared 3 structurally distinct generative approaches (a VAE, a GAN, and a GC) but did not evaluate tree-based synthesizers, which have also shown promise on small tabular datasets. A systematic comparison of tree-based generators on VST data is a useful direction for future work.

In assessing the 3 real clinical datasets, not all features shared the same correlations with response. For example, treatment with foscarnet had the top positive correlation with response (0.20) in the Westmead dataset, a primarily adult cohort, but had a small negative correlation with response (−0.06) in the ACES dataset, a primarily pediatric dataset, and a small negative correlation with response (−0.017) in the Cincinnati mixed-age dataset. This indicates a potential area of further investigation into the combined relationship between age, treatment with foscarnet, and VST response. Prior studies of giving foscarnet for CMV infections after transplant have not focused on the role of age, where most studies include either only adults or only children. However, foscarnet has been shown to be effective in both adults and children [47–49].

Beyond the correlation analysis, we also identified each feature’s contribution to the model’s predictive ability using the Shapley method. The Shapley analysis allows us to assess if certain features are contributing significantly more to the predictive ability of the model and therefore should be given greater attention in our interpretation of the model. We chose to use the Shapley method as it is a standard in the machine learning field. While the individual Shapley values for each feature do not imply that an increase or decrease in that feature (i.e., an increase or decrease in viral load) would result in the patient being more or less likely to respond to a VST infusion, it is interesting to note which features had larger absolute Shapley values relative to others. Notably, all but one of the BMT donor type categories had positive Shapley values, with the exception of mismatched unrelated donor having a negative Shapley value. This indicates an area of further investigation into how HLA matching for BMT may also play a broader role in the outcomes of downstream cellular therapy. In a future study, it may also be beneficial to include details of the HLA matching between the BMT donor and the VST product. This information was not available for all 3 cohorts included in this study, but further details of the HLA matching between the donor, product, and patient should be utilized when available. Additionally, we acknowledge that the overall small values may imply that the model is identifying artifacts in the dataset, so we do not make broad statements about how these features may behave in other VST cohorts.

To select features for this study, we included only variables that were common among the 3 VST cohorts. If a feature was available in only 1 or 2 of the cohorts, we excluded it from the study rather than imputing it for the remaining cohort(s). Other methods of feature selection may have resulted in a different set of features and ultimately a different generative model performance and binary classifier performance. One such method would have been eliminating variables based on their Spearman correlations with response, only keeping variables with a correlation above some absolute value threshold. We could also utilize a multivariate selection method, such as minimum redundancy maximum relevance, wherein features are selected if they have strong correlations with response but weak correlations with each other [50]. This would theoretically eliminate features that are perhaps contributing the same predictive quality to the model and are therefore redundant.

Future directions to improve the accuracy of our existing approach include training on more datasets to improve the model’s performance across different cohorts and inclusion of additional features. The mean F1-score of over 70% across the 3 datasets implies a use case of stratifying patients in a future trial to split them into candidates for third-party VSTs versus additional antiviral pharmacotherapy (if available), which could potentially increase RRs for VST therapy by avoiding administration of VSTs to patients who are unlikely to benefit. However, it is important to note that there are limitations to the model’s predictive ability and there are likely edge cases not yet identified for which our model may not perform accurately. As VST data continue to be generated, the model should be retrained to reflect any new trends in VST features. In some VST datasets, the RRs are imbalanced, and if needed, resampling with replacement could be used to overcome issues of imbalance when training the generative model. In our current model, the performance does not appear to be impacted when tested on imbalanced datasets. Our model could in the future consider more features, including the specific third-party VST products and/or selection criteria, which undoubtedly contribute to RRs, for example, the degree of HLA match between the BMT donor and the VST product. Computational modeling, including genAI approaches, have been proposed for treatment selection to improve clinical outcomes [51,52]. Our approach therefore provides a promising solution to predict treatment responses to third-party VST treatment in immunocompromised patients with viral infections post-BMT. We report discrimination, using ROC-AUC and precision–recall curves, but did not formally assess model calibration, as the withheld real-data test sets are too small to estimate calibration reliably. Calibration should be assessed before any prospective clinical application of this model.

In future studies, there is potential to model VST response as a multiclass problem instead of a binary problem, wherein the predicted outcomes are nonresponse, partial response, and complete response. For the purposes of this study, we utilized a binary method to avoid concerns about introducing additional complexity to the modeling problem before proving that the VST data could be appropriately modeled at all. Additionally, we do not include data pertaining to the VST products themselves; however, another future study may focus on the ranking of individual products as most to least likely to result in a response for any given patient.

In summary, we have shown that a combined generative and classification approach can be used to accurately generate and predict patient clinical response after third-party VST infusion. Synthetic data generated preserved correlations between features and modestly improved the performance of machine learning models to predict patient response to VST therapy. Our classifier model accurately predicts patient response, achieving a median F1-score over of at least 70% across all 3 cohorts. We identified top clinical features contributing to the model’s predictive power and confirmed their value in the third-party VST setting. The method presented in this study is a novel approach to patient stratification, which will facilitate optimal patient selection for VST therapy versus continued antiviral use. Importantly, our framework will be generally applicable to building accurate predictive models for patient response, using clinical trial datasets with limited sample sizes.

## Data Availability

The source data and source code used in this study are included in the Supplementary Codes.

## Supplementary Materials

Supplementary 1 Figs. S1 to S7 Supplemental Information Supplementary Codes
