# Supplementary material for: Boosting the Predictive Power of Virus-Specific T-Cell Clinical Trials via Generative Models
Source: Comput Struct Biotechnol J. 2026 Sep 21;35(1):0208. doi: 10.34133/csbj.0208 (PMC13590963; doi:10.34133/csbj.0208)
Supplement: Supplementary file 1 [file csbj.0208.f1.zip › Supplementary Figures.docx]

**Supplementary Figure 1**

Title: Correlations between variables within each clinical dataset.

Description: From left to right, the Spearman correlations in the ACES dataset, Cincinnati dataset, and Westmead dataset. Heatmap is colored by the Spearman correlation values, where more negative values are in red and more positive values are in blue.

**Supplementary Figure 2**

Title: Training a predictive classifier on different quantities of VAE-generated data results in similar accuracy metrics.

Description: Results of training the custom neural network classifier on varying quantities of ACES-trained-VAE-synthesized data and tested on corresponding withheld ACES, Cincinnati, and Westmead records. The quantities tested are 500, 5000, and 50000 records. Note, only the subset of records passing biological rules are used in the NN training. (A) Box-and-whisker plots displaying the results from 10-fold cross validation for loss (binary cross-entropy), accuracy, recall, precision, F1, and negative predictive value. (B) Precision recall curves. Colored by which quantity of data was used to train the NN.

**Supplementary Figure 3**

Title: Average similarity score across all variables in the dataset after generation using varying numbers of layer and nodes in the VAE.

Description: The best performing VAEs had the medium number of layers and either a large or medium number of nodes.

**Supplementary Figure 4**

Title: Accuracy, precision, and recall scores of various predictive machine learning models when tested on ACES, Cincinnati, and Westmead datasets.

Description: Most models share similar performance, between 0.6 and 0.8 in the three accuracy metrics. Loss is above 1.0 in most models and test cases.

**Supplementary Figure 5**

Title: Non-synthetic data have reduced accuracy when used to train a predictive classifier.

Description: The ACES dataset was split into training and testing for the predictive classifier. (A) Box-and-whisker plots of accuracy metrics of the NN classifier when trained on synthetic versus non-synthetic data. (B) Precision recall curves for the NN classifier when trained on synthetic versus non-synthetic data. (C) Shapley values of each feature in the NN when trained on the non-synthetic ACES data.

**Supplementary Figure 6**

Title: Quality scores for each variable after synthesis by the 3 methods.

Description: VAE and GAN quality scores for each variable are similar, GC quality scores are vastly different in several variables when compared to the results of the VAE and GAN. After applying the biological rules to the synthetic data, the majority of GC-generated records are removed.

**Supplementary Figure 7**

Title: Foundation model for small datasets has varying performance on clinical VST data

Description: The accuracy, precision, and recall are presented for the TabPFN model when trained and tested on different clinical datasets. Each subplot represents the model when it is trained on a portion of the ACES, Cincinnati, and Westmead datasets. Color indicates which dataset is used for testing. When testing on the same clinical dataset that is used for training we ensure that the data were correctly split into training and testing sets to avoid data leakage and bias.
