# Supplementary material for: Boosting the Predictive Power of Virus-Specific T-Cell Clinical Trials via Generative Models
Source: Comput Struct Biotechnol J. 2026 Sep 21;35(1):0208. doi: 10.34133/csbj.0208 (PMC13590963; doi:10.34133/csbj.0208)
Supplement: Supplementary file 1 [file csbj.0208.f1.zip › SupplementaryFigure1.pdf]

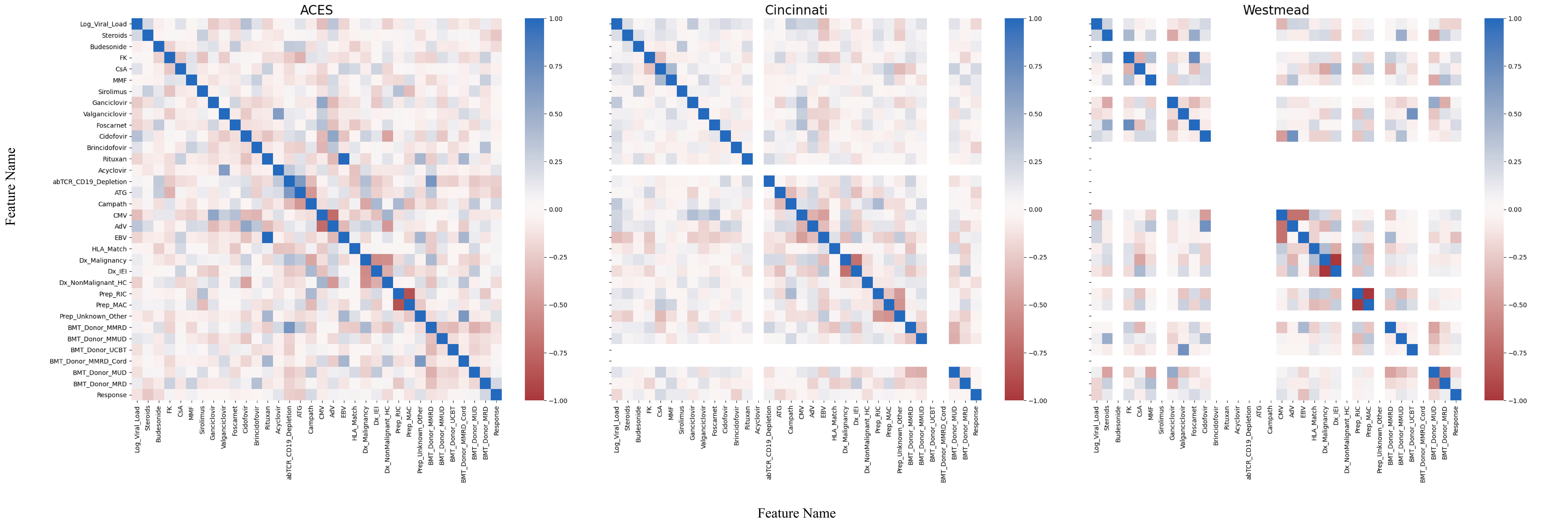
ACES
Cincinnati
Westmead
Feature Name
Feature Name
Log_Viral_Load
Steroids
Budesonide
FK
CsA
MMF
Sirolimus
Ganciclovir
Valganciclovir
Foscarnet
Cidofovir
Brincidofovir
Rituxan
Acyclovir
abTCR_CD19_Depletion
ATG
Campath
CMV
AdV
EBV
HLA_Match
Dx_Malignancy
Dx_IEI
Dx_NonMalignant_HC
Prep_RIC
Prep_MAC
Prep_Unknown_Other
BMT_Donor_MMRD
BMT_Donor_MMUD
BMT_Donor_UCBT
BMT_Donor_MMRD_Cord
BMT_Donor_MUD
BMT_Donor_MRD
Response
1.00
0.75
0.50
0.25
0.00
−0.25
−0.50
−0.75
−1.00
