# Supplementary material for: Boosting the Predictive Power of Virus-Specific T-Cell Clinical Trials via Generative Models
Source: Comput Struct Biotechnol J. 2026 Sep 21;35(1):0208. doi: 10.34133/csbj.0208 (PMC13590963; doi:10.34133/csbj.0208)
Supplement: Supplementary file 1 [file csbj.0208.f1.zip › SupplementaryFigure2_new.pdf]

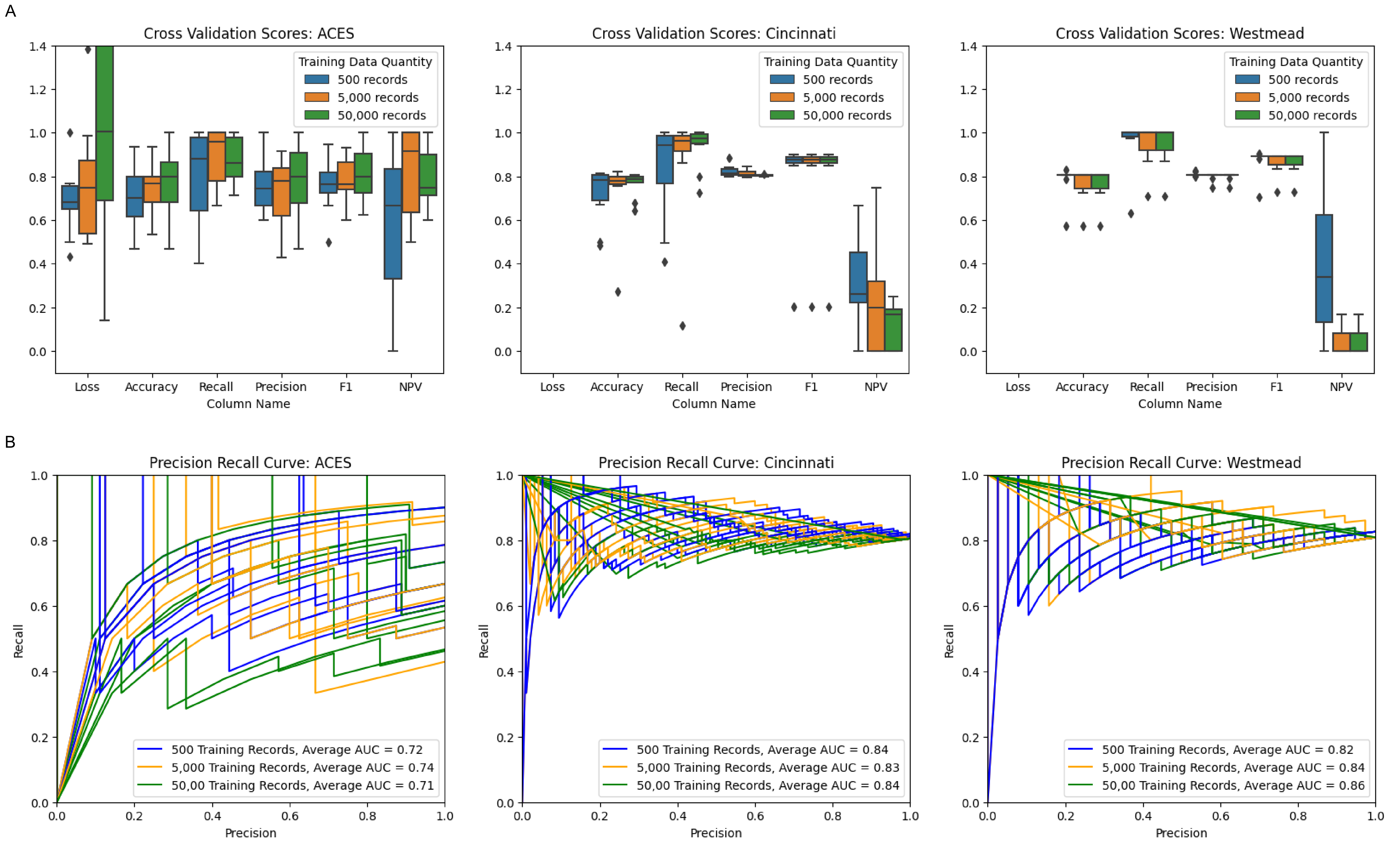
A
Cross Validation Scores: ACES
Cross Validation Scores: Cincinnati
Cross Validation Scores: Westmead
Training Data Quantity
500 records
5,000 records
50,000 records
Loss
Accuracy
Recall
Precision
F1
NPV
Column Name
B
Precision Recall Curve: ACES
Precision Recall Curve: Cincinnati
Precision Recall Curve: Westmead
500 Training Records, Average AUC = 0.72
5,000 Training Records, Average AUC = 0.74
50,00 Training Records, Average AUC = 0.71
500 Training Records, Average AUC = 0.84
5,000 Training Records, Average AUC = 0.83
50,00 Training Records, Average AUC = 0.84
500 Training Records, Average AUC = 0.82
5,000 Training Records, Average AUC = 0.84
50,00 Training Records, Average AUC = 0.86
Recall
Precision
