# Supplementary material for: Boosting the Predictive Power of Virus-Specific T-Cell Clinical Trials via Generative Models
Source: Comput Struct Biotechnol J. 2026 Sep 21;35(1):0208. doi: 10.34133/csbj.0208 (PMC13590963; doi:10.34133/csbj.0208)
Supplement: Supplementary file 1 [file csbj.0208.f1.zip › SupplementaryFigure3_new.pdf]

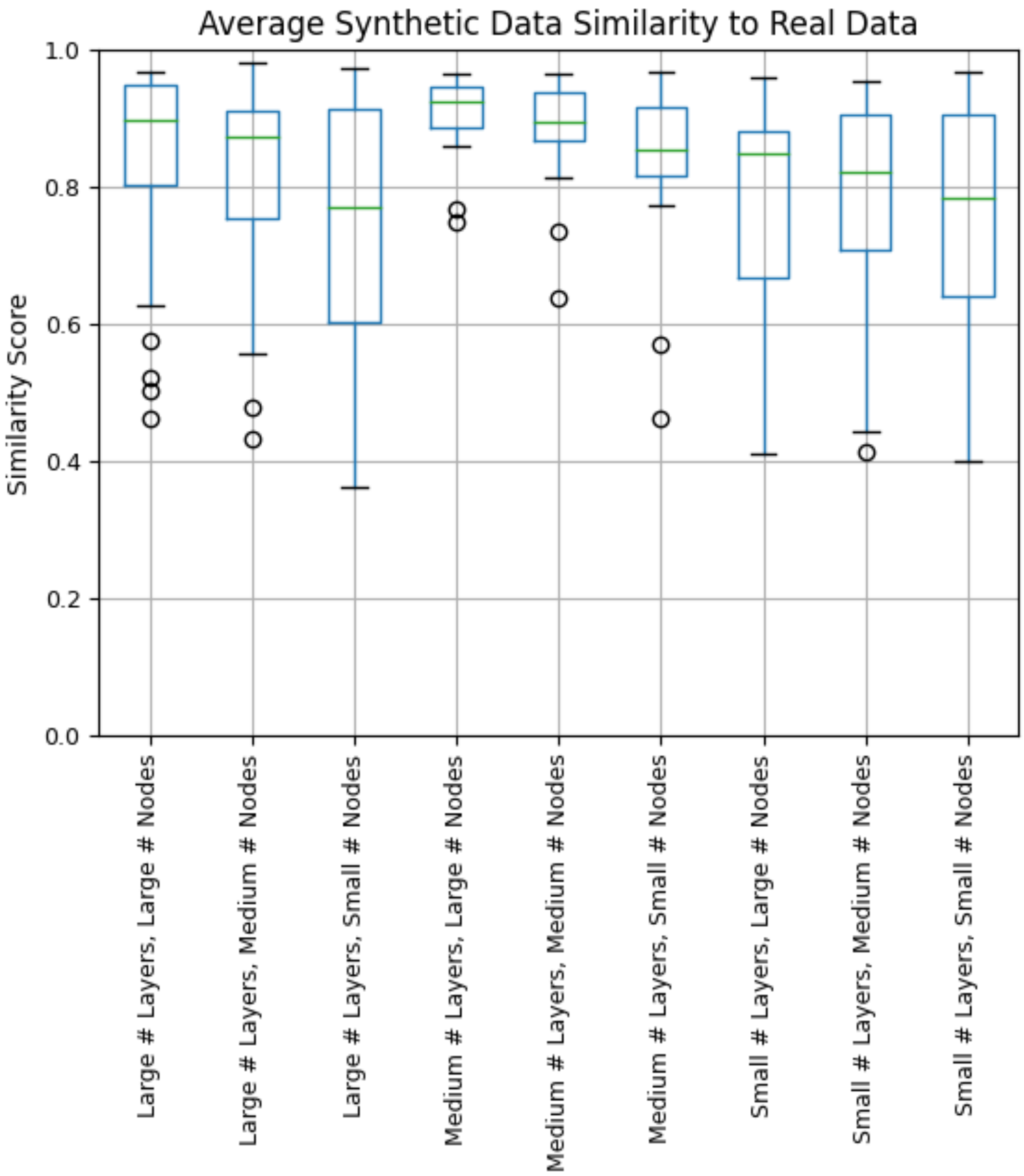

Average Synthetic Data Similarity to Real Data
Similarity Score
1.0
0.8
0.6
0.4
0.2
0.0
Large # Layers, Large # Nodes
Large # Layers, Medium # Nodes
Large # Layers, Small # Nodes
Medium # Layers, Large # Nodes
Medium # Layers, Medium # Nodes
Medium # Layers, Small # Nodes
Small # Layers, Large # Nodes
Small # Layers, Medium # Nodes
Small # Layers, Small # Nodes
