# Supplementary material for: Boosting the Predictive Power of Virus-Specific T-Cell Clinical Trials via Generative Models
Source: Comput Struct Biotechnol J. 2026 Sep 21;35(1):0208. doi: 10.34133/csbj.0208 (PMC13590963; doi:10.34133/csbj.0208)
Supplement: Supplementary file 1 [file csbj.0208.f1.zip › SupplementaryFigure4_new.pdf]

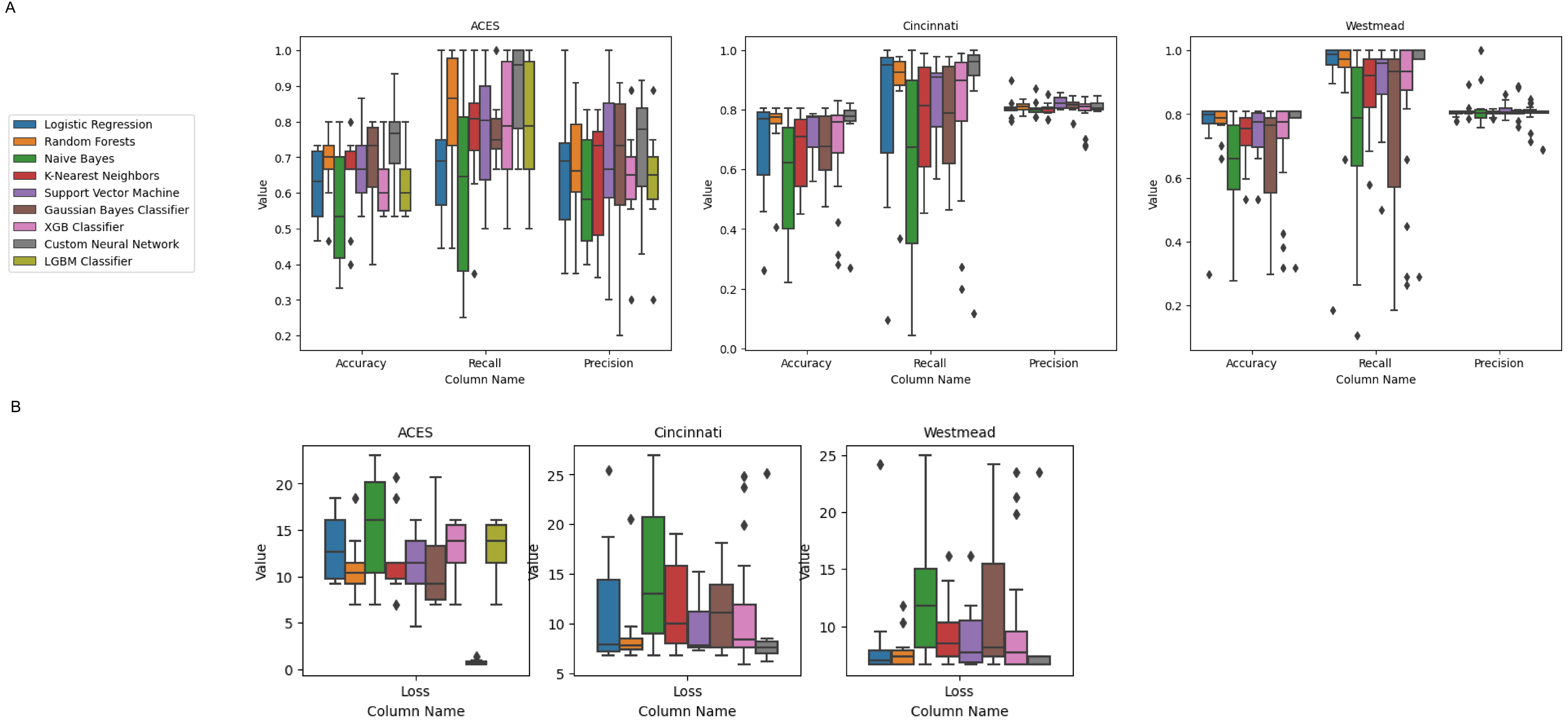

A
Logistic Regression
Random Forests
Naive Bayes
K-Nearest Neighbors
Support Vector Machine
Gaussian Bayes Classifier
XGB Classifier
Custom Neural Network
LGBM Classifier
ACES
Cincinnati
Westmead
Value
Accuracy
Recall
Precision
Column Name
B
ACES
Cincinnati
Westmead
Value
Loss
Column Name
