# Supplementary material for: Boosting the Predictive Power of Virus-Specific T-Cell Clinical Trials via Generative Models
Source: Comput Struct Biotechnol J. 2026 Sep 21;35(1):0208. doi: 10.34133/csbj.0208 (PMC13590963; doi:10.34133/csbj.0208)
Supplement: Supplementary file 1 [file csbj.0208.f1.zip › SupplementaryFigure5_new.pdf]

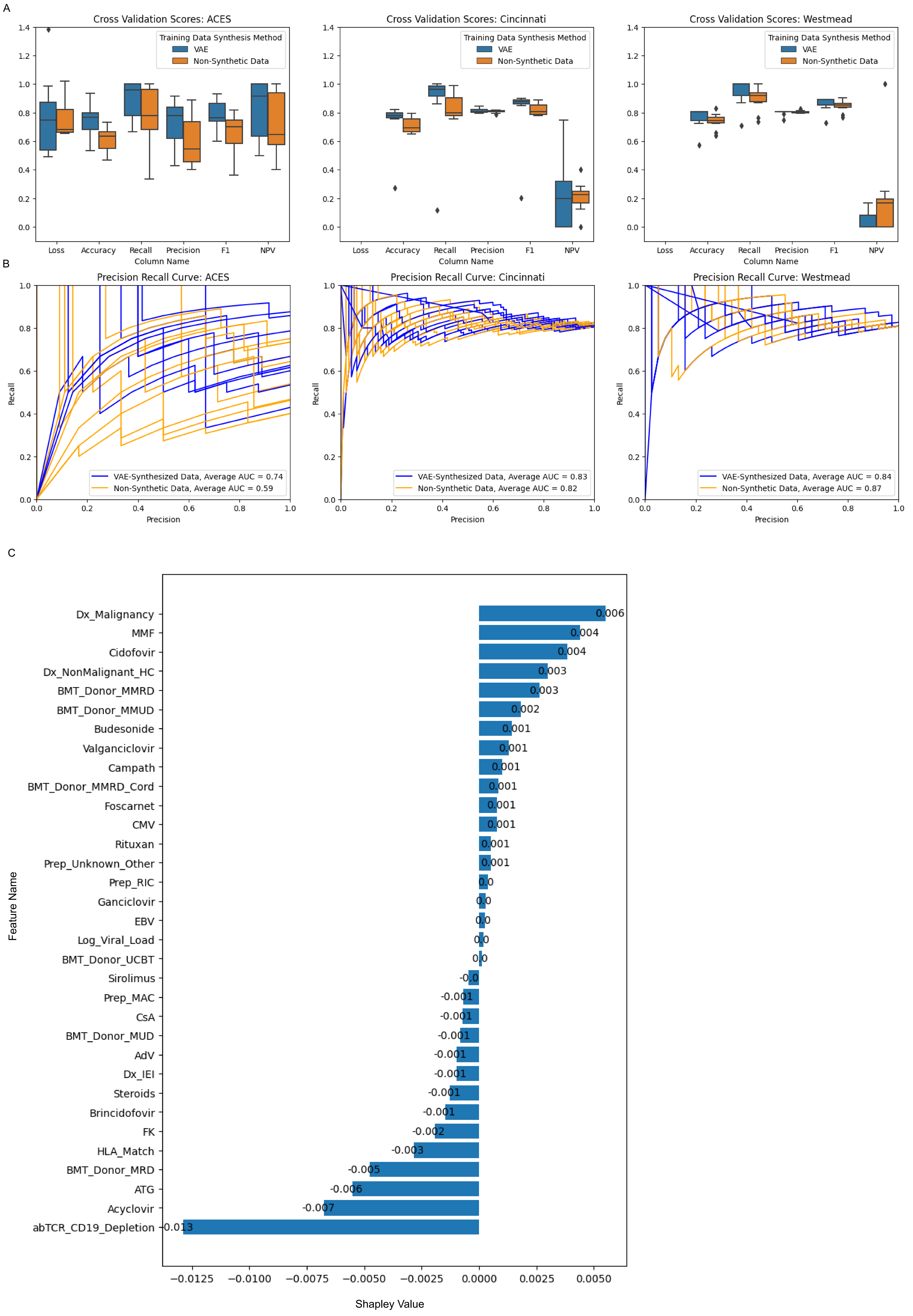
A
Cross Validation Scores: ACES
Cross Validation Scores: Cincinnati
Cross Validation Scores: Westmead
Training Data Synthesis Method
VAE
Non-Synthetic Data
Loss
Accuracy
Recall
Precision
F1
NPV
Column Name
B
Precision Recall Curve: ACES
Precision Recall Curve: Cincinnati
Precision Recall Curve: Westmead
Recall
Precision
VAE-Synthesized Data, Average AUC = 0.74
Non-Synthetic Data, Average AUC = 0.59
VAE-Synthesized Data, Average AUC = 0.83
Non-Synthetic Data, Average AUC = 0.82
VAE-Synthesized Data, Average AUC = 0.84
Non-Synthetic Data, Average AUC = 0.87
C
Feature Name
Shapley Value
Dx_Malignancy 0.006
MMF 0.004
Cidofovir 0.004
Dx_NonMalignant_HC 0.003
BMT_Donor_MMRD 0.003
BMT_Donor_MMUD 0.002
Budesonide 0.001
Valganciclovir 0.001
Campath 0.001
BMT_Donor_MMRD_Cord 0.001
Foscarnet 0.001
CMV 0.001
Rituxan 0.001
Prep_Unknown_Other 0.001
Prep_RIC 0.0
Ganciclovir 0.0
EBV 0.0
Log_Viral_Load 0.0
BMT_Donor_UCBT 0.0
Sirolimus -0.0
Prep_MAC -0.001
CsA -0.001
BMT_Donor_MUD -0.001
AdV -0.001
Dx_IEI -0.001
Steroids -0.001
Brincidofovir -0.001
FK -0.002
HLA_Match -0.003
BMT_Donor_MRD -0.005
ATG -0.006
Acyclovir -0.007
abTCR_CD19_Depletion -0.013
