# Supplementary material for: Boosting the Predictive Power of Virus-Specific T-Cell Clinical Trials via Generative Models
Source: Comput Struct Biotechnol J. 2026 Sep 21;35(1):0208. doi: 10.34133/csbj.0208 (PMC13590963; doi:10.34133/csbj.0208)
Supplement: Supplementary file 1 [file csbj.0208.f1.zip › SupplementaryFigure6_new.pdf]

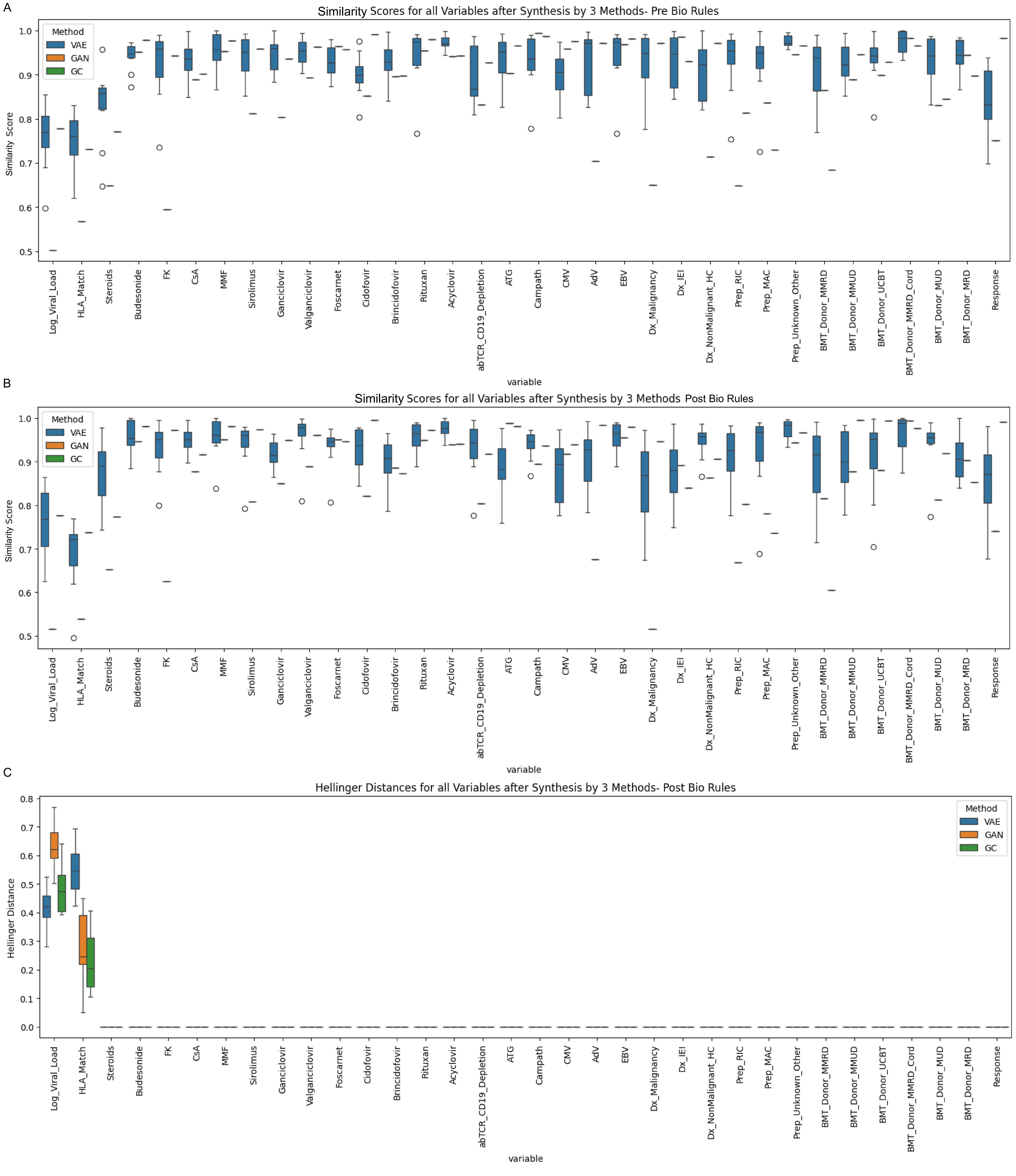
A
Similarity Scores for all Variables after Synthesis by 3 Methods- Pre Bio Rules
Similarity Score
Method
VAE
GAN
GC
1.0
0.9
0.8
0.7
0.6
0.5
Log_Viral_Load
HLA_Match
Steroids
Budesonide
FK
CsA
MMF
Sirolimus
Ganciclovir
Valganciclovir
Foscarnet
Cidofovir
Brincidofovir
Rituxan
Acyclovir
abTCR_CD19_Depletion
ATG
Campath
CMV
AdV
EBV
Dx_Malignancy
Dx_IEI
Dx_NonMalignant_HC
Prep_RIC
Prep_MAC
Prep_Unknown_Other
BMT_Donor_MMRD
BMT_Donor_MMUD
BMT_Donor_UCBT
BMT_Donor_MMRD_Cord
BMT_Donor_MUD
BMT_Donor_MRD
Response
variable
B
Similarity Scores for all Variables after Synthesis by 3 Methods Post Bio Rules
Similarity Score
Method
VAE
GAN
GC
variable
C
Hellinger Distances for all Variables after Synthesis by 3 Methods- Post Bio Rules
Hellinger Distance
0.8
0.7
0.6
0.5
0.4
0.3
0.2
0.1
0.0
Method
VAE
GAN
GC
variable
