# Supplementary material for: Boosting the Predictive Power of Virus-Specific T-Cell Clinical Trials via Generative Models
Source: Comput Struct Biotechnol J. 2026 Sep 21;35(1):0208. doi: 10.34133/csbj.0208 (PMC13590963; doi:10.34133/csbj.0208)
Supplement: Supplementary file 1 [file csbj.0208.f1.zip › SupplementaryFigure7.pdf]

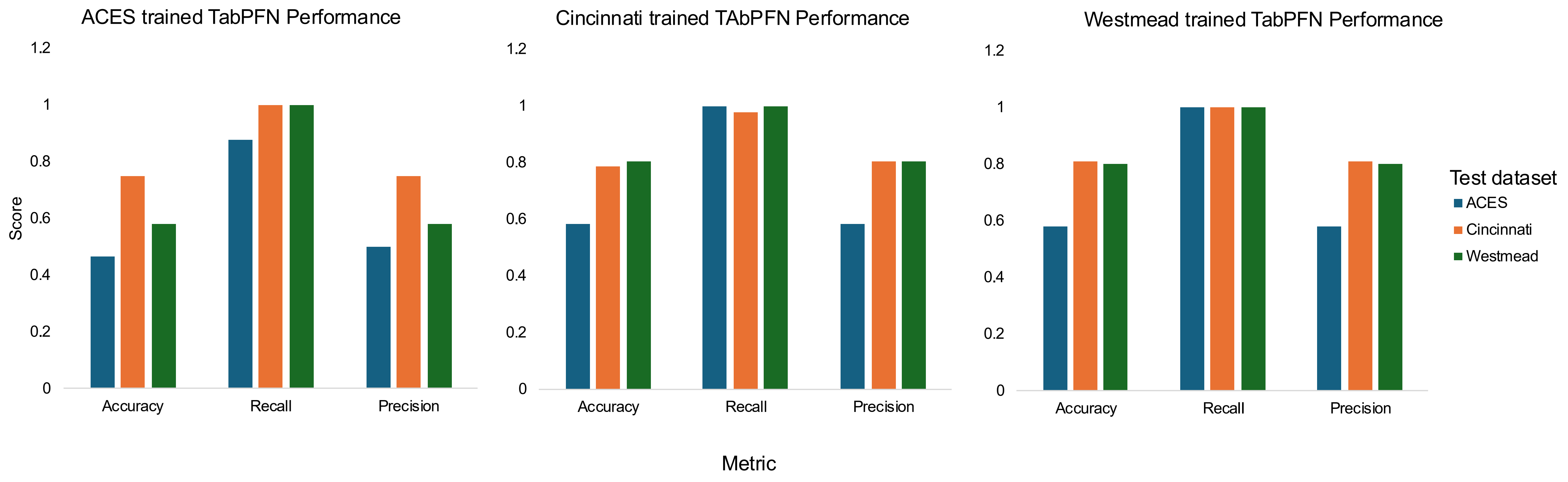
ACES trained TabPFN Performance
Cincinnati trained TAbPFN Performance
Westmead trained TabPFN Performance
Score
1.2
1
0.8
0.6
0.4
0.2
0
Accuracy
Recall
Precision
Metric
Test dataset
ACES
Cincinnati
Westmead
